# Point of care testing for improving risk- benefit ratio of aspirin and warfarin

**DOI:** 10.1186/1755-8166-7-S1-I54

**Published:** 2014-01-21

**Authors:** Harsh Sheth, Emma Northwood, Faye Elliott, Michael Jackson, Mauro Santibanez Koref, John Tyson, Ann Daly, Jonathan O’Halloran, Jayesh Sheth, Frenny Sheth, Keyur Parikh, D Timothy Bishop, John Burn

**Affiliations:** 1 Institute of Genetic Medicine, Newcastle University, International Centre for Life, Newcastle upon Tyne, UK; 2 Leeds Institute of Molecular Medicine, St. James Hospital, Beckett Street, Leeds, UK; 3 QuantuMDx Ltd., International Centre for Life, Newcastle upon Tyne, UK; 4 Institute of Cellular Medicine, Medical School, Newcastle University, Newcastle upon Tyne, UK; 5 FRIGE’s Institute of Human Genetics, FRIGE House, Satellite, Ahmedabad, India; 6 CIMS Hospital, Nr. Shakun Mall, Sola, Ahmedabad, India

## 

The increase in identification of putative biomarkers and opportunities to develop tailored treatments are due to emergence of *omics* technologies. Application of pharmacogenetic knowledge with the help of quick and cheap companion diagnostics in the primary care setting is expected to deliver improved treatment and reduced heathcare costs. Warfarin and aspirin are the two most widely prescribed drugs for preventing cardiovascular diseases. Long term aspirin use has also been shown to reduce risk, recurrence and mortality from colorectal cancer. However, they both have narrow therapeutic windows and several genetic polymorphisms have been noted to influence their dose and efficacy. We therefore have launched two collaborative projects: first, to study the genetics of warfarin safety in the Gujarati Indian population and second, to identify further polymorphisms that modulates aspirin’s colorectal cancer chemopreventive efficacy. Understanding the impact of polymorphisms on dose and efficacy for these drugs would lead to development of a combined panel of markers that would predict accurate therapeutic dose with minimal risk for adverse reactions. These markers will be deployed at the point of care settings using a novel handheld genotyping device which will use disposable microfluidic cassettes and silicon nanowires currently developed by QuantuMDx. Results, future work, opportunities and barriers will be examined.

